# MiR-29a Knockout Aggravates Neurological Damage by Pre-polarizing M1 Microglia in Experimental Rat Models of Acute Stroke

**DOI:** 10.3389/fgene.2021.642079

**Published:** 2021-03-15

**Authors:** Fangfang Zhao, Haiping Zhao, Junfen Fan, Rongliang Wang, Ziping Han, Zhen Tao, Yangmin Zheng, Feng Yan, Yuyou Huang, Lei Yu, Xu Zhang, Xiaolong Qi, Lianfeng Zhang, Yumin Luo, Yuanwu Ma

**Affiliations:** ^1^Institute of Cerebrovascular Diseases Research and Department of Neurology, Xuanwu Hospital of Capital Medical University, Beijing, China; ^2^Beijing Geriatric Medical Research Center and Beijing Key Laboratory of Translational Medicine for Cerebrovascular Diseases, Beijing, China; ^3^Key Laboratory of Human Disease Comparative Medicine, National Health Commission of China (NHC) and Beijing Engineering Research Center for Experimental Animal Models of Human Critical Diseases, Institute of Laboratory Animal Science, Chinese Academy of Medical Sciences, Peking Union Medicine College, Beijing, China; ^4^Neuroscience Center, Chinese Academy of Medical Sciences, Beijing, China

**Keywords:** Ischemic stroke, miR-29a, microglia, astrocyte, glutamate

## Abstract

**Objective:**

By exploring the effects of miR-29a-5p knockout on neurological damage after acute ischemic stroke, we aim to deepen understanding of the molecular mechanisms of post-ischemic injury and thus provide new ideas for the treatment of ischemic brain injury.

**Methods:**

miR-29a-5p knockout rats and wild-type SD rats were subjected to transient middle cerebral artery occlusion (MCAO). miR-29a levels in plasma, cortex, and basal ganglia of ischemic rats, and in plasma and neutrophils of ischemic stroke patients, as well as hypoxic glial cells were detected by real-time PCR. The infarct volume was detected by TTC staining and the activation of astrocytes and microglia was detected by western blotting.

**Results:**

The expression of miR-29a-5p was decreased in parallel in blood and brain tissue of rat MCAO models. Besides, miR-29a-5p levels were reduced in the peripheral blood of acute stroke patients. Knockout of miR-29a enhanced infarct volume of the MCAO rat model, and miR-29a knockout showed M1 polarization of microglia in the MCAO rat brain. miR-29a knockout in rats after MCAO promoted astrocyte proliferation and increased glutamate release.

**Conclusion:**

Knockout of miR-29a in rats promoted M1 microglial polarization and increased glutamate release, thereby aggravating neurological damage in experimental stroke rat models.

## Background

Ischemic stroke is considered a disease that causes significant morbidity and mortality on a global scale. Although some progress has been made in animal models and clinical treatments, there are many gaps in research to understand the mechanisms and influencing factors of stroke. The inflammatory response of the brain to ischemia is another area of interest in stroke. However, it is not fully understood how various inflammatory factors affect the brain during and after cerebral ischemia. Studies have shown that the inflammatory response is an important pathophysiological basis for ischemic cerebrovascular disease. The first step in the inflammatory response in the brain is the activation and polarization of microglia, which in turn initiates the release of effector molecules and activation of other immune cells. The transition of microglia from a quiescent to an activated state is a dynamic process. Activated microglia are divided into two phenotypes based on their surface markers and functions, M1 (pro-inflammatory/harmful) and M2 (anti-inflammatory/protective), both of which are Iba1 highly expressed in their cell membranes (Kigerl et al., 2009; Zhang et al., 2017; Yao and Zu, 2020). M1 is activated through the classical pathway, expressing CD16 and iNOS on cell membranes, and secretes inflammatory factors such as tumor necrosis factor-α (TNF-α) and interleukin-6 (IL-6), which exacerbate post-stroke brain injury (Iadecola and Anrather, 2012; Xiong et al., 2016; Kim and Bae, 2017). M2 is activated by an alternative pathway and expresses the characteristic markers CD206 and Arg1 on the cell membrane. It has a strong phagocytic ability and can engulf damaged nerve cells and tissue fragments to repair trauma and release some neuroprotective factors.

MicroRNA (miRNA) is a class of small, non-coding, single-stranded RNA molecules of approximately 19–25 nucleotides in size that are widespread in eukaryotes. It is involved in the regulation of a number of important physiological and pathological processes in the human body through complete or incomplete complementary matching of the 3′-untranslated region (3′-UTR) of its downstream target gene, resulting in the degradation of the target mRNA or inhibition of its protein translation (Vos et al., 2019). Recent studies have shown that the expression of ischemia-associated miRNAs is significantly altered in the serum of patients with cerebral ischemia and that such changes are strongly associated with stroke and other disease progression. Several miRNAs are involved in the regulation of cell proliferation, apoptosis, and embryonic development, which are especially important for the development of cerebrovascular diseases (Weiner, 2018). Recent studies revealed that the expression level of miR-29 family members altered in rodent models of focal ischemia and clinical stroke patients (Jeyaseelan et al., 2008; Tan et al., 2009). The miR-29 family includes miR-29a, miR-29b, and miR-29c, which have been found to play important roles in a variety of diseases (Lagos-Quintana et al., 2001; Shi et al., 2017). miR-29a, which is enriched in brain tissue, is a good candidate miRNA for brain disease research. miR-29a expression levels in the serum of patients with acute cerebral infarction and their association with prognosis have not been investigated. Therefore, by exploring the effects of miR-29a knockout on neurological damage after acute ischemic stroke, we aimed to deepen our understanding of the molecular mechanisms of post-ischemic brain injury and thus provide new ideas for the treatment of ischemic stroke.

## Materials and Methods

Our manuscript completely adheres to the AHA Journals’ implementation of the Transparency and Openness Promotion (TOP) Guidelines.

### Animal Model of Focal Cerebral Ischemia

Rats used in this research were bred in standard cages provide with standard food and water *ad libitum* in stand SPF animal facility. All animal experiments were approved by the Animal Care and Use Committees of the Institute of Laboratory Animal Science of Peking Union Medical College (ZLF18004). Focal cerebral ischemia was induced by transient middle cerebral artery occlusion (MCAO), as previously described (Zhao et al., 2019). Briefly, rats were anesthetized by enflurane inhalation; the right common carotid artery was exposed, and the external carotid artery was ligated and transected. A 3-cm length of 3/0 nylon filament with a tip that was rounded by silicone (tip diameter: 0.38 mm) was inserted through the puncture in the external carotid artery and gently advanced along the internal carotid artery to a point 18–19 mm from the bifurcation. The thread was removed 1.5 h after ischemia to allow reperfusion. In the sham group, rats were exposed to the same surgical procedure but without filament insertion. To confirm occlusion, the local cerebral blood flow in the area supplied by the middle cerebral artery was evaluated by a PeriFlux System 5000 transcranial LDF laser Doppler (Perimed, Järfälla, Sweden). Body temperature was monitored continuously with a rectal probe and maintained at 37.0 ± 0.5°C during the procedure using a heating lamp.

### Generation of miR-29a Knockout Rat

**FIGURE 1:**
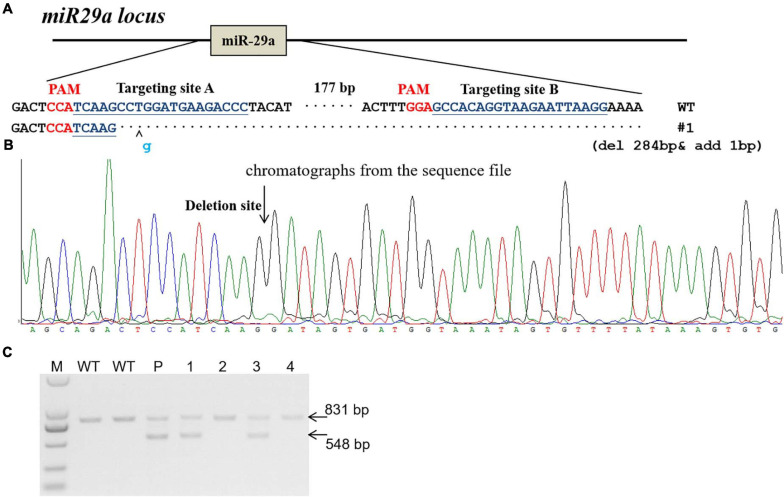
Generation of miR-29a knockout rat using CRISPR/Cas9 system. **(A)** sgRNA used for targeting. Two sgRNA targeting sites (targeting site A and targeting site B) were designed for miR-29a deletion in rats. The PAM sequence is highlighted in red. The deleted DNA fragment were shown in **(A)**. **(B)** Chromatographs from the sequence file. The deletion site was indicated with arrow. **(C)** PCR identification of CRISPR/Cas9-mediated site-specific cleavage of the endogenous miR-29a locus. P, F0 (parents); 1–4, number of F1 generation. Wild-type PCR product band was 831 bp, and the miR-29a knockout PCR brand was 548 bp.

The miR-29a knockout rats were produce by CRISPR/Cas9 system as described before (Ma et al., 2014). In brief, two sgRNAs were designed to target the upstream and downstream of miRNA gene locus. sgRNA target sites were indicated in Figure 1. *In vitro* transcripted sgRNA and Cas9 mRNA were mixed and microinjected into fertilized rat eggs and then transferred to pseudopregnant SD rats to be carried to parturition. Primers (Rat miR29a-check-S: AGGGAAGAATGCAGCAAGTGACT; Rat miR29a-check-AS: ATGTTTCCTGCTCCTCTCACATTG) were used for miR-29a knockout rat genotyping.

### Animal Grouping

One set of experiments for the expression levels of miR-29a at different time points. A total of 36 male rats were randomly divided into the following four groups: (1) wild-type rats received sham surgery (*n* = 6); (2) miR-29a knockout rats received sham surgery (*n* = 6); (3) wild-type rats received MCAO surgery (*n* = 12); (4) miR-29a knockout rats received MCAO surgery (*n* = 12). Rats in the sham group underwent the same procedure as those in other groups but without suture insertion. Six rats in groups 3 and 4 were used for brain infarction and edema analyses, and six were used for molecular detection and western blot assessment. Six 2 mm-thick coronal sections were cut 3, 5, 7, 9, 11, and 13 mm posterior to the olfactory bulb. Brain slices #1–#3 were used for enzyme-linked immunosorbent assay, and slices #4–#6 were used for western blotting.

### Clinical Patient Selection

The human project was approved by the Committee of Institutional Review Board of Capital Medical University, Beijing, China. Written and oral informed consent was obtained from patients or legal representatives according to the Helsinki Declaration and the Helsinki Declaration was carried out during the human studies. This prospective study analyzed 40 ischemic stroke patients in the emergency department or neurology ward of Xuanwu Hospital, Capital Medical University, China, from March to December 2017. Twenty-seven control volunteers who did not have any focal neurological deficit and antecedents of central nervous system (CNS) disease were recruited from Medical Examination Center of Xuanwu Hospital. Volunteers in the control group were matched for age and sex to the patients in acute stroke groups. Ischemic stroke was diagnosed by neurologists based on the patient’s medical history, physical examination, and radiologic diagnosis on admission in accordance with guidelines formulated in 2014 (Dharap et al., 2009). The inclusion criteria consisted: 1. first ischemic stroke and admission within 6 h after symptom onset; 2. National Institute of Health stroke scale (NIHSS) < 25 points; 3. sudden occurrence of a focal neurological deficit and exclude hemorrhage using CT scan; 4. adequate access to patient information. The exclusion criteria included: 1. recurrent stroke; 2. hematological system diseases, malignant tumors, renal, or liver failure; 3. having history of mental disorders, severe dementia, or coronary artery disease; 4. other diseases affecting the hemogram.

### Blood Preparation and miRNA Extraction

Blood samples from stroke patients were collected within 6 h after stroke symptom onset. Blood sample of patients (4 ml × 2) was collected into tubes containing ethylenediaminetetraacetic acid (EDTA) by venipuncture before any other therapies. Blood samples were processed according to the following procedures: firstly, the blood sample was centrifuged at 200 g for 10 min at 4°C immediately and got plasma, and then the plasma was fractionated into RNase/DNase-free vials and stored at −80°C for the further laboratory test. Secondly, diluting the blood cells with 8 ml normal saline (NS) and adding the blood cells to the surface of the lymphocyte separation medium (Tian Jin Hao Yang Biological Manufacture Co., Ltd., China) slowly in two 15 ml centrifuge tube. After the tubes were centrifuged at 400 g for 20 min at 20°C, the lymphocytes can be separated and saved. At last, erythrocytes were dissociated with erythrocyte lysing solution and the remaining neutrophils were saved. Total RNA in neutrophils and lymphocytes was extracted using TRIzol method according to the manufacturer’s protocol and stored at −80°C for further laboratory test.

### Determination of Infarct Volume

Rats were deeply anesthetized 24 h after MCAO and brains were quickly removed and cut into six 2-mm-thick sections. Then they were stained with 1.5% 2,3,5-triphenyltetrazolium chloride. The border between the infarct and healthy tissue was outlined using Image-Pro Plus analysis software (Media Cybernetics, San Diego, CA, United States). Infarct and edema volumes were expressed as a percentage of the contralateral area for each section. Infarct volume percentage (%) = (normal cerebral hemisphere volume-infarct cerebral hemisphere volume)/normal cerebral hemisphere volume × 100%. Edema volume percentage(%) = (infarct cerebral hemisphere volume-normal cerebral hemisphere volume)/normal cerebral hemisphere volume × 100%.

### Quantitative Real-Time Polymerase Chain Reaction

Total RNA in plasma was extracted using TRIzol LS reagent (Invitrogen, Carlsbad, CA, United States) and RNA in neutrophils and lymphocytes was extracted using TRIzol reagent (Invitrogen, Carlsbad, CA, United States) according to the manufacturer’s protocol. A total RNA (300 ng) was reverse transcripted to cDNA using the Superscript III reverse transcriptase kit (Invitrogen, Carlsbad, CA, United States). After the first-strand cDNA was synthesized from total RNA, the Gene Amp PCR System 9700 (Applied Biosystems) was used for the TaqMan based quantitative real-time polymerase chain reaction (qRT-PCR) assays. The primers and probes of the miR-29a-5p and U6 endogenous controls for miRNA assays were purchased from Shanghai Bioligo Technology Co., Ltd.: the PCR primers for hsa-miR-29a-5p were 5′-GGG ACT GAT TTC TTT TGG T-3′ and 5′-GTG CGT GTC GTG GAG TCG-3′; the PCR primers for mmu-miR-29a-5p were 5′-GGG ACT GAT TTC TTT TGG T-3′ and 5′-CAG TGC GTG TCG TGG A3′, the PCR primers for U6 were 5′-GCT TCG GCA GCA CAT ATA CTA AAA T-3′ and 5′-CGC TTC ACG AAT TTG CGT GTC AT-3′; the PCR primers for β-actin were 5′-GTA CCA CCA TGT ACC CAG GC-3′ and 5′-AAC GCA GCT CAG TAA CAG TCC-3′. Real-time PCR was performed according to the manufacturer’s protocols. Relative gene expression was normalized and expressed as fold change relative to that of U6 and calculated via a 2^–Δ^ ^Δ^ ^*CT*^ method.

### Western Blot Analysis

Rats were deeply anesthetized 24 h after MCAO, intracardially perfused with cold PBS, and their brains removed and homogenized in lysis buffer containing protease inhibitors with further sonication and centrifugation at 18,000 × *g* for 30 min. Total protein content in the supernatant was quantified using a bicinchoninic acid assay kit (Pierce, Rockford, IL, United States). Sodium dodecyl sulfate polyacrylamide gel electrophoresis and western blotting were carried out as previously described (Yin et al., 2006) using antibodies against CD206 (Abcam 64693, Cambridge, MA, United States), Arg1 (Cell Signaling Technology 9819, Danvers, MA, United States), CD16 (Abcam 109223), iNOS (Abcam 3523), GFAP (Santa Cruz Biotechnology 33673, Santa Cruz, CA, United States). Following three times washing in Tris-buffered saline with Tween-20, membranes were incubated with horseradish peroxidase (HRP)-conjugated secondary antibodies (1:5000; Abgent, San Diego, CA, United States) for 1 h at room temperature; immunoreactivity was visualized with an enhanced chemiluminescence reagent kit (Millipore, Billerica, MA, United States). The integrated density value was calculated with Quantity One software and normalized to β-actin (Santa Cruz Biotechnology).

### Determination of Glutamic Acid in Brain Tissue by Mass Spectrometry

Take the sample stored at −80°C for 30 min at −20°C, and then place it in the refrigerator at 4°C to melt it; weigh 25 mg of each sample and put it into EP tube; add two small steel balls and 800 μl precipitant (methanol: acetonitrile: pure water = 2:2:1) into each EP tube; grind in a grinder (50 Hz, 4 min); crush the cells and then ultrasonic the sample in ice bath. After centrifugation for 15 min (25,000*g*, 4°C), 500 μl of supernatant was taken; 500 μl supernatant was added into freeze-drying machine; 500 μl 10% methanol solution was added into ice bath ultrasound for 10 min (power 80 Hz), and centrifuged for 15 min (25,000*g*, 4°C); the supernatant was detected on the LC-MS/MS thermo license II liquid phase SCIEX 5500, which is detected by MRM mode. The data is processed by multiquant software (SCIEX, Framingham, MA, United States), and the absolute content of the target compound in the sample is calculated.

### Statistical Analysis

All statistical analysis was performed using the Graph-Pad Prism 7 software (Graph Pad Software Inc., San Diego, CA, United States). Values in the text are presented as means ± SEM. Independent samples *t*-test was used for two-group comparisons. The one way analysis of variance (ANOVA) was used for comparison among several quantitative variables. Differences were considered statistically significant at ^∗^*p* < 0.05, ^∗∗^*p* < 0.01, and ^∗∗∗^*p* < 0.001.

## Results

### MiR-29a-5p Expression Decreased in Blood and Brain Tissue After Ischemic Stroke

**FIGURE 2:**
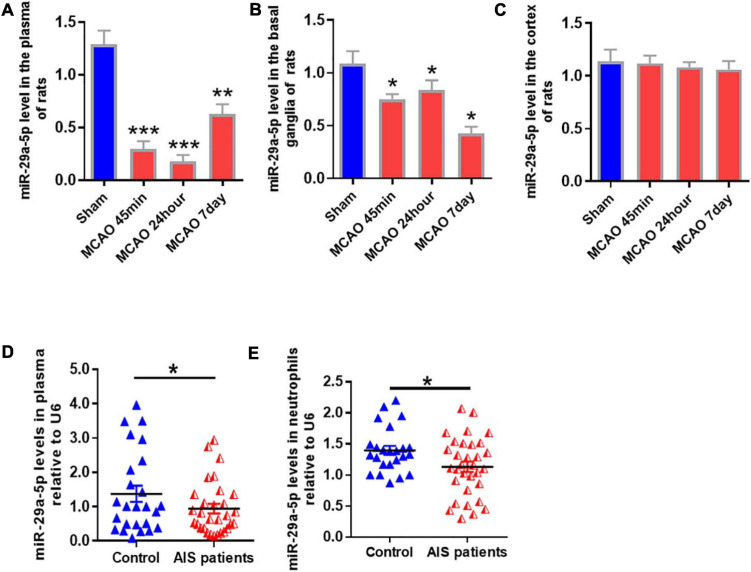
MiR-29a-5p expression in blood and brain tissue of rats and peripheral blood of patients after ischemic stroke. **(A–C)** MiR-29a-5p expression levels in **A** plasma; **(B)** basal ganglla; and **(C)** cortex (*n* = 6) of MCAO rats and sham group (*n* = 6) were detected by real-time PCR. **(D,E)** MiR-29a-5p levels were reduced in plasma **(D)** and neutrophils **(E)** in acute stroke patients (*n* = 40) and control group (*n* = 27). Data represent mean ± SEM. *p < 0.05 compared to control; **p < 0.01 compared to control; ***p < 0.001 compared to control. MCAO, middle cerebral artery occlusion; AIS, acute ischemic stroke.

To determine the expression of miR-29a-5p in circulating blood and brain tissue, we examined miR-29a-5p level in the blood and brain tissue including basal ganglia and cortical following MCAO-induced focal cerebral ischemia of rats at 45 min, 24 h, and 7 days post-reperfusion. We found that the expression levels of miR-29a-5p dramatically decreased in the plasma (Figure 2A, *p* < 0.001) and basal ganglia area (Figure 2B, *p* < 0.05) at 45 min following ischemia, and continued to decrease at 24 h and 7 days post-reperfusion, compared with sham group. However, there was no significant difference of miR-29a-5p levels in the cortex area (Figure 2C), Therefore, the expression of miR-29a-5p was decreased in the brain tissue as well as circulating blood in rat MCAO models.

### MiR-29a-5p Levels Were Reduced in the Neutrophils of Acute Stroke Patients

A total of 40 ischemic stroke and 27 healthy volunteers were enrolled in this study. There was no statistically significant difference in age, sex, or risk factors, including a history of diabetes, hypertension, or hypercholesterolemia (Li et al., 2018). After acute cerebral infarction, neutrophils are rapidly elevated in reactivity. Therefore, we examined miR-29a-5p expression in peripheral plasma and neutrophils from all stroke patients and healthy controls. Our results showed that compared with healthy controls, miR-29a-5p expression level in plasma (Figure 2D, *p* < 0.05) and the neutrophils (Figure 2E, *p* < 0.05) significantly decreased within 6 h after stroke onset.

### MiR-29a-5p Knockout Enhanced Infarct Volume in Rats After MCAO

**FIGURE 3:**
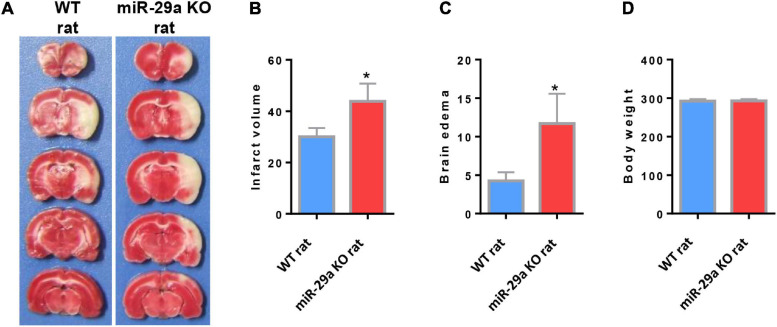
MiR-29a-5p knockout enhanced infarct volume and edema volume in MCAO rats after day 3. **(A)** Coronal sections representing infarcts in wild-type rats and miR-29a knockout rats. **(B)** Bar graph calculating the infarct volume. **(C)** Bar graphs for calculating brain edema volume. **(D)** Bar graph for calculating rat body weight. Data represent mean ± SEM. *n* = 6 per group.*p < 0.05 compared to control. MCAO, middle cerebral artery occlusion.

In order to evaluate the role of miR-29a-5p on neurological damage, we further characterized the brain infarct volume aftermiR-29a-5p knockout. We found that the infarct volume significantly increased in the miR-29a-5p knockout group at 3 days after MCAO compared with the wild-type controls (Figures 3A,B, *p* < 0.05). Similar to the infarct volume measurement results, we found that the edema formation of ipsilateral hemisphere in miR-29a-5p knockout rats was significantly higher than that in the control rat after MCAO (Figures 3A,C, *p* < 0.05). However, miR-29a-5p knockout did not influence the body weight following stroke (Figure 3D). Hence, these data confirmed that miR-29a-5p knockout increased infarct volume in rats after MCAO.

### MiR-29a-5p Knockout Exhibited M1 Shift of Microglia in the Brain of Rats

**FIGURE 4:**
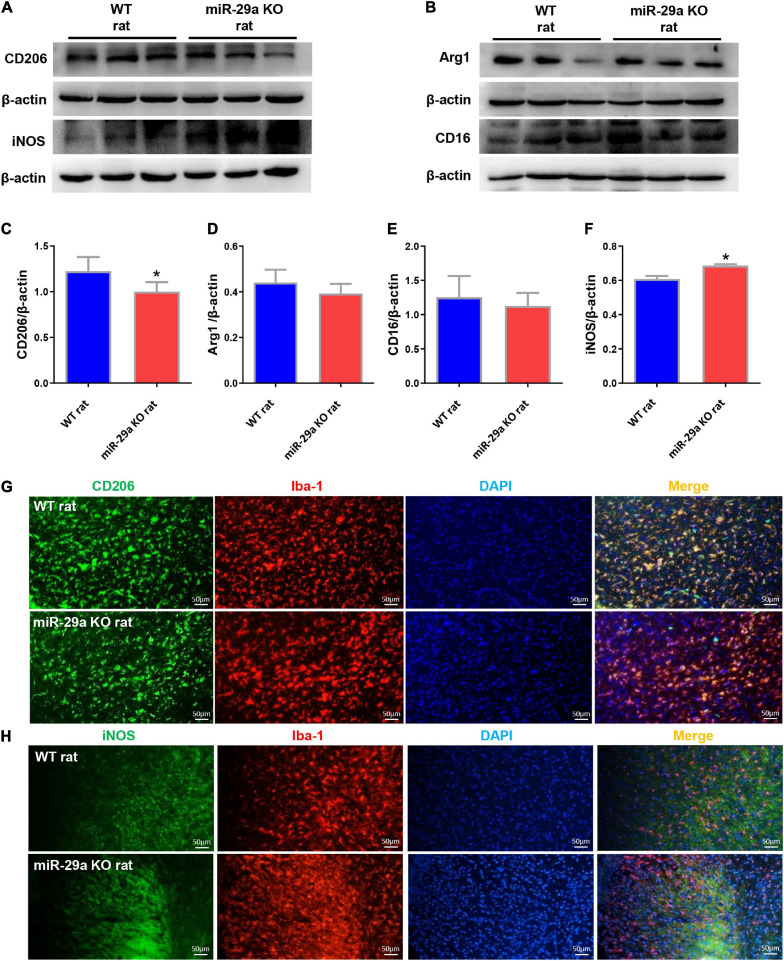
Knockout of miR-29a-5p exhibited M1 polarization of microglia in rat brain. **(A,B)** Western blot detection of microglia M1 and M2 marker changes in the brains of wild-type rats and miR-29a-5p knockout rats. **(A)** CD206, iNOS **(B)** Arg1, CD16. **(C–F)** Bar graphs of marker changes in the brains of wild-type rats and miR-29a-5p knockout rats. **(C)** CD206, **(D)** Arg1, **(E)** CD16, **(F)** iNOS. **(G,H)** Representative double immunofluorescence staining for CD206 (green) or iNOS (green), and Iba-1 (red) markers. **(G)** CD206, **(H)** iNOS. *p < 0.05. Arg1, arginase 1; iNOS, inducible nitric oxide synthase.

Microglial changes are highly dynamic, proliferating extensively in the first 2 weeks of cerebral infarction and aggregating around the lesion. We studied the effect of miR-29a-5p knockout on microglial cell polarization in brain tissue reperfused for 3 days after ischemic stroke. Western blot was used to test the expression of neural markers, mainly including microglial M2 markers, CD206 and Arg1, microglial M1 markers CD16 and iNOS (Figures 4A,B). Double immunofluorescence staining for iNOS or CD206 and Iba-1 (microglial marker) was performed after MCAO (Figures 4G,H). The results showed that miR-29a-5p knockout significantly decreased the protein levels of M2 markers CD206 (Figures 4C,G, *p* < 0.05) while increased the protein levels of M1 markers iNOS (Figures 4F,H, *p* < 0.05) in brain tissues. But there was no significant difference between Argl and CD16 (Figures 4D,E, *p* > 0.05). Therefore, acute stroke induced microglia were converted to an anti-inflammatory phenotype compared to the wild-type group, represented by iNOS/Iba1-positive cells in the miR-29a-5p knockout group.

### MiR-29a-5p Knockout Promoted Astrocyte Proliferation and Increased the Release of Glutamate in Basal Ganglia of Rats After MCAO

**FIGURE 5:**
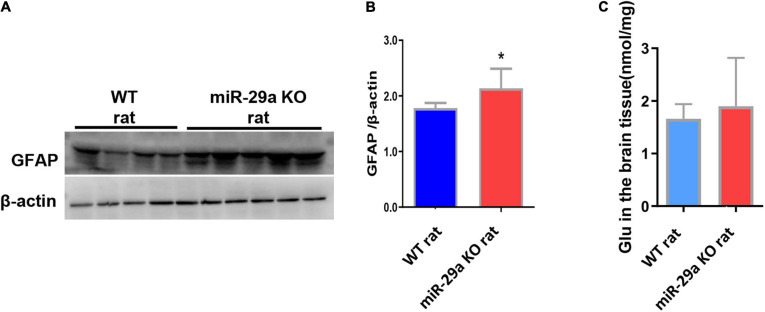
MiR-29a knockout promoted astrocyte proliferation and increased the release of the neurotoxic substance glutamate in basal ganglia of rats after MCAO. **(A)** Detection of protein expression of GFAP in the brains of wild-type rats and miR-29a knockout rats by western blot. The next band corresponds to GFAP. **(B)** Bar graphs of protein expression of GFAP in the brains of wild-type and miR-29a knockout rats. **(C)** Bar graphs of Glu in the brains of wild-type rats and miR-29a knockout rats. Data represent mean ± SEM. *n* = 6 per group. *p < 0.05. GFAP, glial fibrillary acidic protein; Glu, glutamic acid.

After knockout of miR-29a-5p, we observed changes in microglia. In addition, we also observed changes in astrocytes and astrocyte associated glutamate release. We found a significant increase in the number of astrocytes in the miR-29a-5p knockout group after MCAO (Figures 5A,B, *p* < 0.05) and an increase in glutamate release in brain tissue (Figure 5C, *p* < 0.05) compared with the wild-type control group.

## Discussion

Stroke (including ischemic and hemorrhagic stroke) affects 13.7 million people worldwide each year and is the second cause of death (5.5 million deaths per year) (GBD 2016 Neurology Collaborators, 2019; Lindsay et al., 2019). It is also the leading cause of death and disability worldwide. Ischemic stroke accounts for about 71% of all strokes (Katan and Luft, 2018). Numerous recent studies have confirmed that inflammation plays an important role in post-ischemic neuronal injury and enlargement of the infarcted area, and as an important inflammatory cell, the role of microglia in the post-ischemic inflammatory response has received increasing attention (Ritter et al., 2000; Moskowitz et al., 2010; Perry et al., 2010). However, how microglial activation and polarization are regulated after acute ischemic stroke is a question that deserves further discussion.

MicroRNAs are considered as promising targets for stroke therapy. A rapidly growing area of stroke recovery research involves the use of miRNAs, which can manipulate the expression of genes and proteins in the brain after stroke. It is estimated that up to 30% of genes encoding human proteins can be regulated by miRNAs (Ouyang et al., 2013a). miRNAs are thought to play a central role in embryonic development, cell differentiation, and metabolism, and have been used in studies of brain disease therapy. miRNAs are thought to play an important role in ischemic stroke pathology by altering the expression, such as miR-15a, which is involved in ischemic brain injury by inhibiting the anti-apoptotic gene BCL-2 (Yao et al., 2020). A study reported that miR-181a as an antitumor drug applied after MCAO decreased NF-κB expression, increased BCL-2 levels, reduced infarct size, and had beneficial effects on long-term recovery in mice (Xu et al., 2015). Although these results are promising, miRNA research is still relatively new and presents its challenges. However, despite these obstacles, miRNAs represent a promising therapeutic area that may have a substantial impact on the treatment of ischemic stroke. Altered expression of miRNAs, including miR-29 family members, in a rodent model of focal ischemia and clinical stroke patients, were identified by spectrographic analysis and RT-PCR (Jeyaseelan et al., 2008; Tan et al., 2009). The miR-29 family consists of three members (a, b, and c) (Ouyang et al., 2013b), with low expression of miR-29c in in some diseases (Zhang et al., 2011; Kriegel et al., 2012). Clinically, miR-29b was identified as a novel circulating biomarker for stroke prognosis (Wang et al., 2015). In animal models, brain-targeted knockout of miR-29a causes neurological deficits, particularly hippocampal region-specific neuronal cell death (Roshan et al., 2014).

We first demonstrated that miR-29a expression was reduced in the peripheral blood of acute stroke patients. To the best of our knowledge, this is the first time to show that circulating miR-29a levels decreased in AIS patients. We also found that miR-29a was reduced in both blood and brain tissue in a rat model of focal cerebral ischemia. This is consistent with the findings of Jeyaseelan et al. (2008) and Tan et al. (2009). Our results show that miR-29a-5p decreases in the basal ganglia but not the cortex after ischemia in rat brain tissue, which may be associated with fewer branches of cerebrovascular blood supply to the basal ganglia relative to the cortex. Whether circulating miR-29a reflects the response of brain tissue to ischemia is unclear. miR-29a knockout enhances infarct volume in MCAO rats. Clinically, miR-29b is significantly downregulated in stroke patients, and it is negatively correlated with NIHSS score and infarct volume (Wang et al., 2015). However, the clinical significance of the miR-29 family on microglial activation in brain tissue after cerebral ischemia is unclear. We further investigated the effect of miR-29a knockout on microglia, and our study found that rats exhibited M1 shift of microglia in the brain after MCAO. In summary, the results of the present study indicate that miR-29a knockout promotes M1 microglial polarization and exacerbates neurological injury in experimental stroke in rats. Ashley et al. also found that miR-29a/b was associated with microglia polarization and the mechanism of action was mainly through the downregulation of Insulin-like growth factor-1 (IGF-1) and Insulin-like growth factor-1 (CX3CL1) (Fenn et al., 2013).

The importance of miR-29a on the outcome of cerebral ischemia was further confirmed by miR-29a knockout. Similar to previous results, miR-29a knockout enhanced infarct volume and reduced neurogenesis in MCAO rats. A full understanding of the regulatory mechanisms of miRNA networks represents a new direction for stroke research. The miR-29 family exhibits differential regulation and subcellular distribution, and target expression of various proteins such as collagen, transcription factors, and methyltransferases (Liu et al., 2010). Unexpectedly, we discovered a new miR-29a target regulatory network that promotes critical glial cell functions to restore steady-state control after ischemia-reperfusion injury, including maintenance of glutamate signaling.

Our study also found that miR-29a knockout promotes astrocyte proliferation and increases the release of the neurotoxic substance glutamate in rats after MCAO. The excitotoxicity of glutamate plays a key role in neuronal death after ischemic stroke. Classically, the uptake of glutamate from the extracellular space and the release of glutamate into neurons are two major processes that astrocytes undergo in the CNS. Under inflammatory conditions in the CNS, astrocytes may lose one or both of these functions, leading to the accumulation of extracellular glutamate, which eventually leads to excitotoxic neuronal death and worsens CNS inflammation (Mahmoud et al., 2019). Emerging evidence suggests that microglial activation triggers astrocyte-mediated regulation of excitatory neurotransmission (Bezzi et al., 2001; Pascual et al., 2012; Macht, 2016). The importance of miR-29 in glutamate uptake after cerebral ischemia was recently reported using AGO CLIP and whole transcriptome profiling. Acute loss of miR-29 may increase brain glutamate levels in response to stroke through a dynamic pattern of network activation (Wang et al., 2010). In our study, it was also found that down-regulation of miR-29a increased glutamate accumulation.

## Conclusion

In summary, this study provides a new approach to the treatment of acute ischemic brain injury by exploring the effects of miR-29a knockout on neuroinflammation after stroke and its underlying mechanisms. We firstly discovered that miR-29a knockout can increase infarct volume and brain edema and aggravate brain injury. Based on this rationale, miR-29a is expected to be a potential biomarker and therapeutic agent for the treatment of acute cerebral ischemia.

## Data Availability Statement

The raw data supporting the conclusions of this article will be made available by the authors, without undue reservation.

## Ethics Statement

The studies involving human participants were reviewed and approved by the Committee of Institutional Review Board of Capital Medical University, Beijing, China. The patients/participants provided their written informed consent to participate in this study. The animal study was reviewed and approved by the Animal Care and Use Committees of the Institute of Laboratory Animal Science of Peking Union Medical College (ZLF18004). Written informed consent was obtained from the individual(s) for the publication of any potentially identifiable images or data included in this article.

## Author Contributions

FZ wrote the manuscript. HZ, JF, RW, ZH, ZT, YZ, FY, YH, LY, XZ, and LZ took part in the experiment and modified it. YL and YM designed and critically revised the manuscript. XQ did the construction work of miR-29a genetic modified rats. All authors contributed to the article and approved the submitted version.

## Conflict of Interest

The authors declare that the research was conducted in the absence of any commercial or financial relationships that could be construed as a potential conflict of interest.
