# Corrigendum: MiR-29a Knockout Aggravates Neurological Damage by Pre-polarizing M1 Microglia in Experimental Rat Models of Acute Stroke

**DOI:** 10.3389/fgene.2021.738582

**Published:** 2021-10-26

**Authors:** Fangfang Zhao, Haiping Zhao, Junfen Fan, Rongliang Wang, Ziping Han, Zhen Tao, Yangmin Zheng, Feng Yan, Yuyou Huang, Lei Yu, Xu Zhang, Xiaolong Qi, Lianfeng Zhang, Yumin Luo, Yuanwu Ma

**Affiliations:** ^1^ Institute of Cerebrovascular Diseases Research and Department of Neurology, Xuanwu Hospital of Capital Medical University, Beijing, China; ^2^ Beijing Geriatric Medical Research Center and Beijing Key Laboratory of Translational Medicine for Cerebrovascular Diseases, Beijing, China; ^3^ Key Laboratory of Human Disease Comparative Medicine, National Health Commission of China (NHC) and Beijing Engineering Research Center for Experimental Animal Models of Human Critical Diseases, Institute of Laboratory Animal Science, Chinese Academy of Medical Sciences, Peking Union Medicine College, Beijing, China; ^4^ Neuroscience Center, Chinese Academy of Medical Sciences, Beijing, China

**Keywords:** ischemic stroke, miR-29a, microglia, astrocyte, glutamate

In the original article, we neglected to include the funder “CAMS Innovation Fund for Medical Sciences (CIFMS), 2019-I2M-1-004” to Xiaolong Qi.

“This project was supported by the National Natural Science Foundation of China (81771413, 82001268, and 81771412) and Beijing Natural Science Foundation Program and the Scientific Research Key Program of the Beijing Municipal Commission of Education (KZ201810025041). Xuanwu Hospital Science Program for Fostering Young Scholars (QNPY2020005). The present work was supported in part by the CAMS Innovation Fund for Medical Sciences (CIFMS) (2017-I2M-3-015; 2019-I2M-1-004 to XQ).”

Also there was a mistake in Figure 3–5 as published. The word “rat” was miswritten as “mice.” The corrected Figure 3–5 appears below.

**FIGURE 3:**
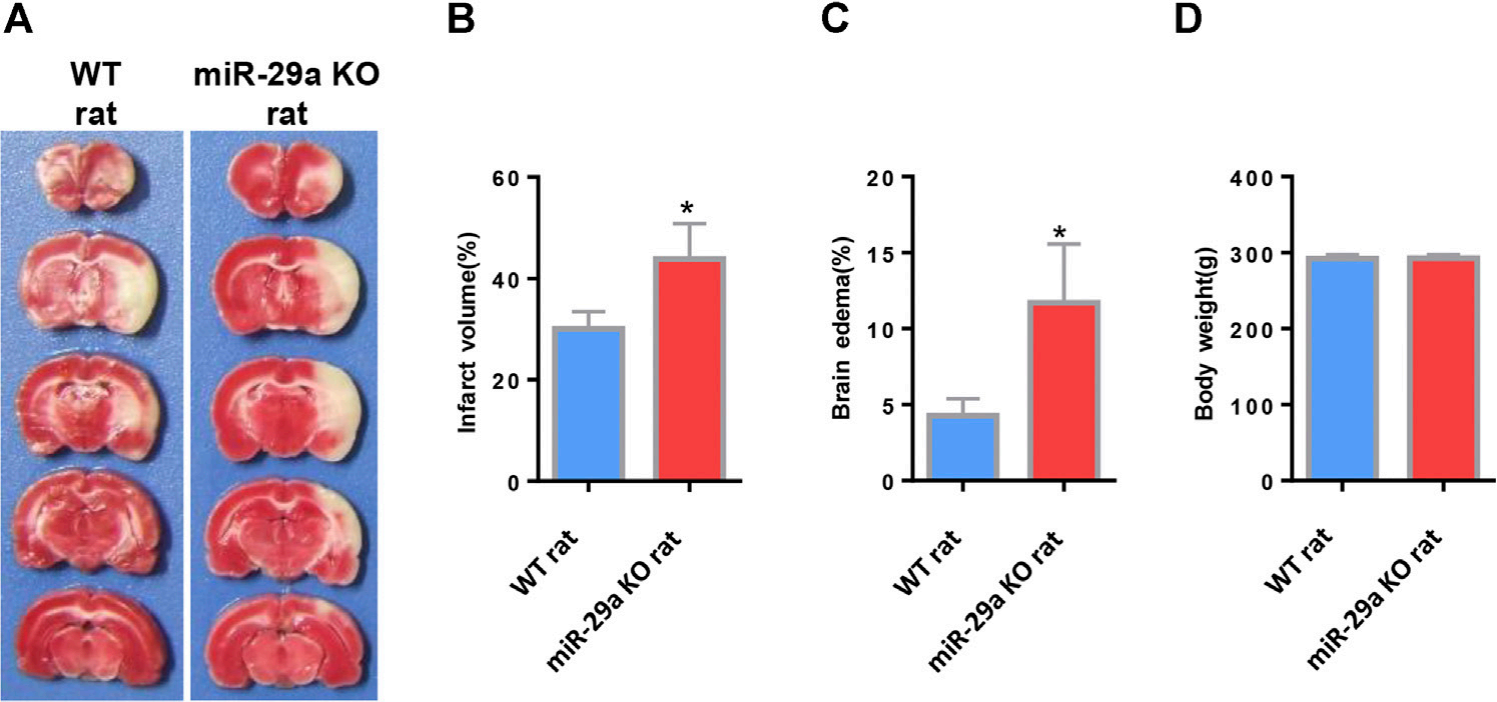
MiR-29a knockout enhanced infarct volume and edema volume in MCAO rats after day 3. **(A)** Coronal sections representing infarcts in wild-type rats and miR-29a knockout rats. **(B)** Bar graph calculating the infarct volume. **(C)** Bar graphs for calculating brain edema volume. **(D)** Bar graph for calculating rat body weight. Data represent mean ± SEM. *n* = 6 per group.*p < 0.05 compared to control. MCAO, middle cerebral artery occlusion.

**FIGURE 4:**
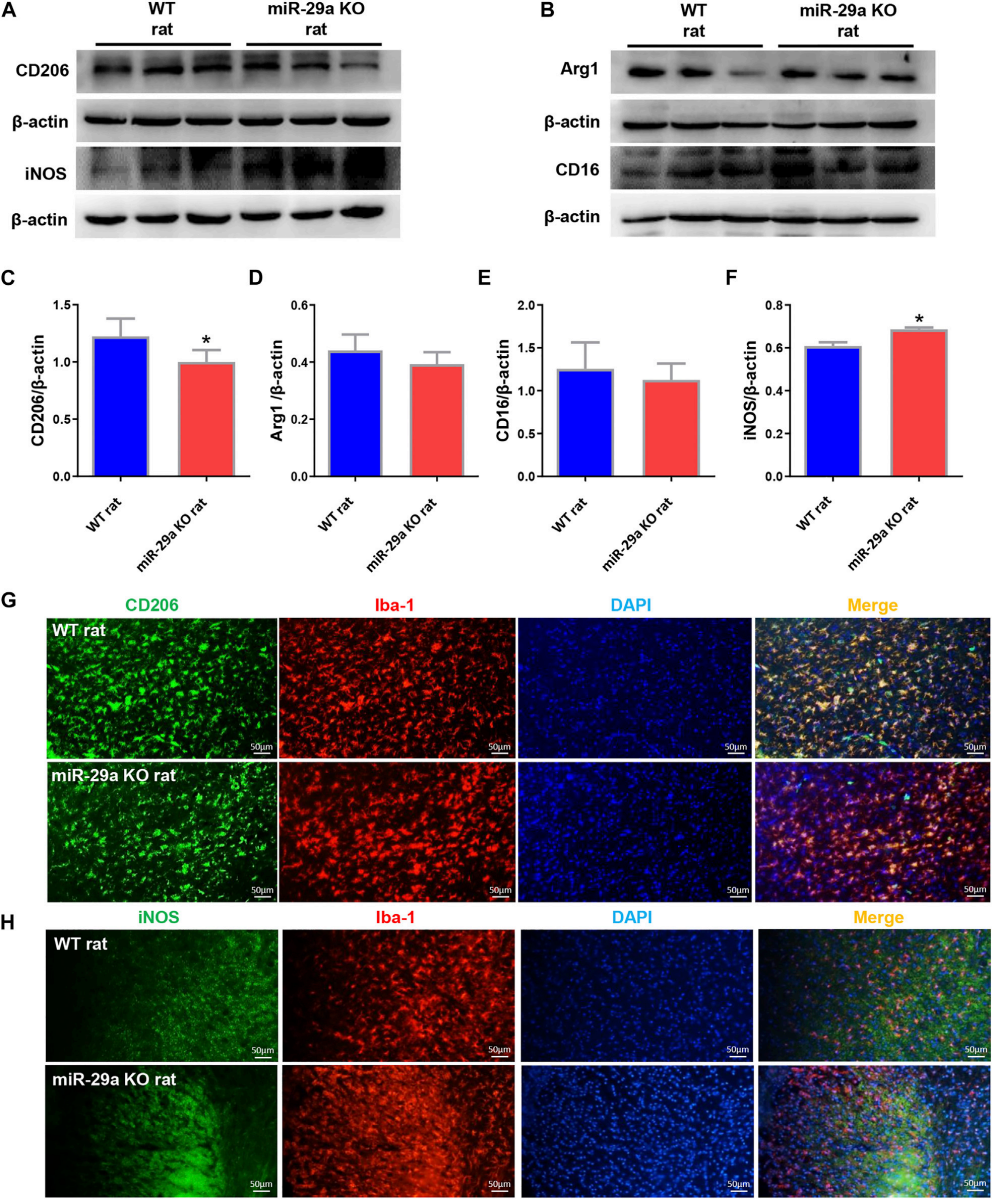
Knockout of miR-29a exhibited M1 polarization of microglia in rat brain. **(A,B)** Western blot detection of microglia M1 and M2 marker changes in the brains of wild-type rats and miR-29a-5p knockout rats. **(A)** CD206, iNOS **(B)** Arg1, CD16. **(C–F)** Bar graphs of marker changes in the brains of wild-type rats and miR-29a knockout rats. **(C)** CD206, **(D)** Arg1, **(E)** CD16, **(F)** iNOS. **(G,H)** Representative double immunofluorescence staining for CD206 (green) or iNOS (green), and Iba-1 (red) markers. **(G)** CD206, **(H)** iNOS. *p < 0.05. Arg1, arginase 1; iNOS, inducible nitric oxide synthase.

**FIGURE 5:**
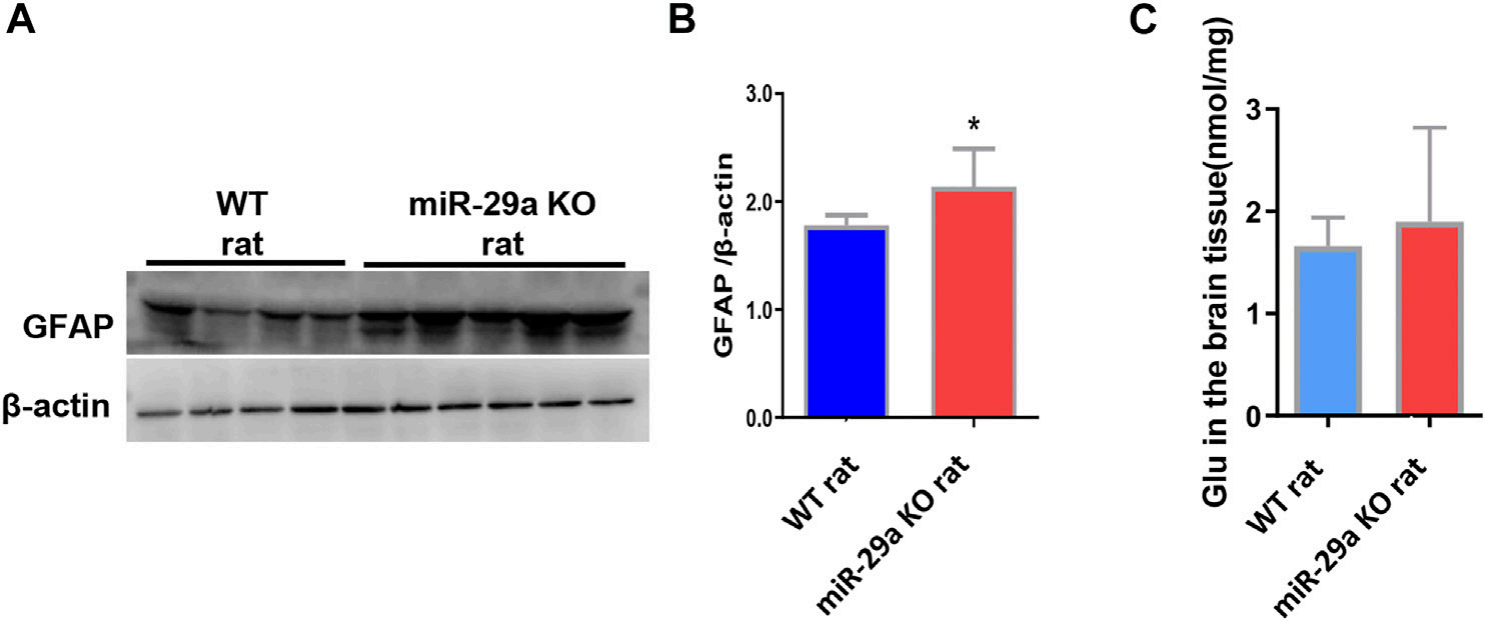
MiR-29a knockout promoted astrocyte proliferation and increased the release of the neurotoxic substance glutamate in basal ganglia of rats after MCAO. **(A)** Detection of protein expression of GFAP in the brains of wild-type rats and miR-29a knockout rats by western blot. The next band corresponds to GFAP. **(B)** Bar graphs of protein expression of GFAP in the brains of wild-type and miR-29a knockout rats. **(C)** Bar graphs of Glu in the brains of wild-type rats and miR-29a knockout rats. Data represent mean ± SEM. *n* = 6 per group. *p < 0.05. GFAP, glial fibrillary acidic protein; Glu, glutamic acid.

Finally, there was an error in authorship. Xiaolong Qi was not included as an author in the published article. Authorlist has been corrected and his contribution has been added to the “Author Contributions.” The corrected statement appears below.

“FZ wrote the manuscript. HZ, JF, RW, ZH, ZT, YZ, FY, YH, LY, XZ, and LZ took part in the experiment and modified it. YL and YM designed and critically revised the manuscript. XQ did the construction work of miR-29a genetic modified rats All authors contributed to the article and approved the submitted version.”

The authors apologize for this error and state that this does not change the scientific conclusions of the article in any way. The original article has been updated.

